# Ecological factors are likely drivers of eye shape and colour pattern variations across anthropoid primates

**DOI:** 10.1038/s41598-022-20900-6

**Published:** 2022-10-15

**Authors:** Juan Olvido Perea-García, Kokulanantha Ramarajan, Mariska E. Kret, Catherine Hobaiter, Antónia Monteiro

**Affiliations:** 1 Department of Biological Sciences, National University of Singapore, Singapore, 117557 Singapore; 2 School of Applied Sciences, Republic Polytechnic, Singapore, Singapore; 3 Institute of Psychology, Cognitive Psychology Unit, Leiden University, Wassenaarseweg 52, 2333 AK Leiden, The Netherlands; 4 Centre for Social Learning and Cognitive Evolution and Scottish Primate Research Group, School of Psychology and Neuroscience, University of St Andrews, Fife, Scotland, UK; 5 Science Division, Yale-NUS College, Singapore, Singapore

**Keywords:** Ecology, Evolutionary ecology

## Abstract

External eye appearance across primate species is diverse in shape and colouration, yet we still lack an explanation for the drivers of such diversity. Here we quantify substantial interspecific variation in eye shape and colouration across 77 primate species representing all extant genera of anthropoid primates. We reassess a series of hypotheses aiming to explain ocular variation in horizontal elongation and in colouration across species. Heavier body weight and terrestrial locomotion are associated with elongated eye outlines. Species living closer to the equator present more pigmented conjunctivae, suggesting photoprotective functions. Irises become bluer in species living further away from the equator, adding to existing literature supporting a circadian clock function for bluer irises. These results shift the current focus from communicative, to ecological factors in driving variation in external eye appearance in anthropoid primates. They also highlight the possibility that similar ecological factors contributed to selection for blue eyes in ancestral human populations living in northern latitudes.

## Introduction

**Figure 1:**
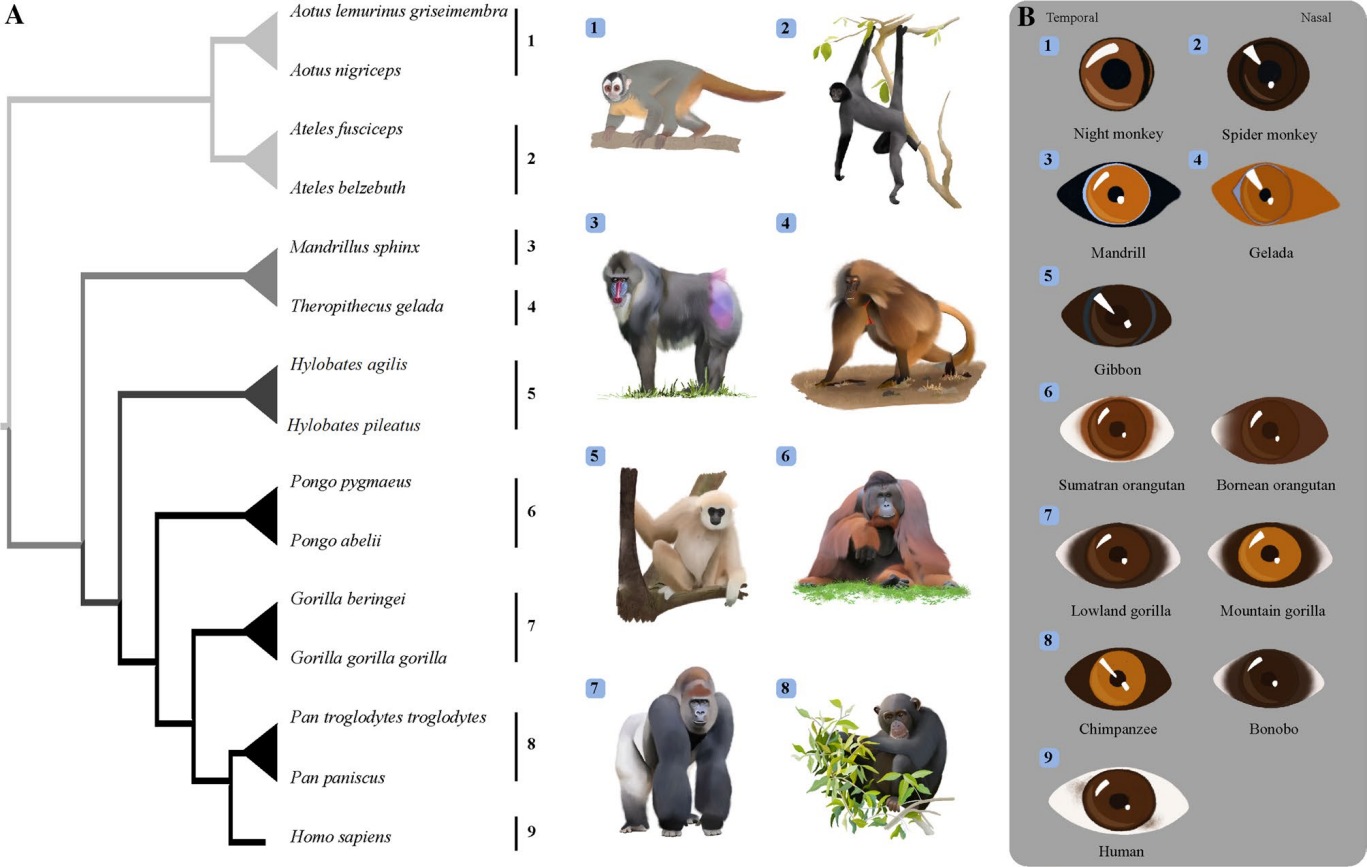
Anthropoid primates are a diverse taxon, including arboreal and terrestrial species with a broad geographical distribution. External eye appearance is also diverse in anthropoid primates. Even very closely related species may have remarkably different patterns of colouration (see pairs of species in 7 or 8). All illustrations by Anupama Prakash.

**Figure 2:**
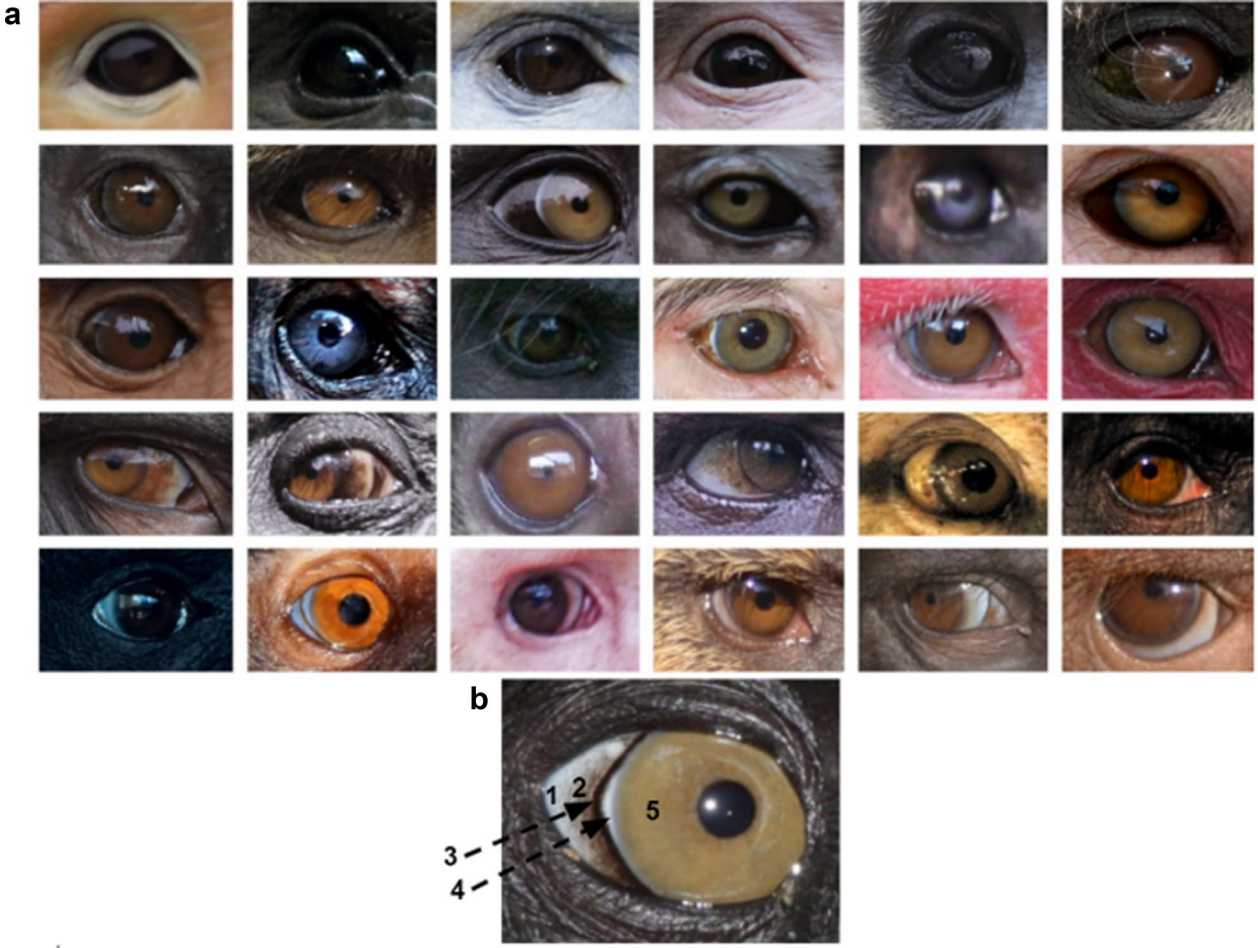
The eyes of anthropoid primates are extremely diverse in shape and colouration. (**a**) Species represented in this panel are, from left to right, top row: *Pygathrix nemaeus, Brachyteles arachnoides, Rhinopithecus roxellana, Rhinopithecus bieti, Hylobates pileatus, Colobus guereza;* second row: *Trachypithecus cristatus, Papio anubis, Mandrillus sphinx, Macaca fascicularis, Ateles belzebuth, Macaca fuscata*; third row: *Piliocolobus badius, Ateles hybridus, Colobus angolensis, Macaca mulatta, Cacajao calvus, Macaca fuscata*; fourth row: *Theropithecus gelada, Gorilla gorilla gorilla, Aotus lemurinus griseimembra, Sapajus apella, Macaca sylvanus, Trachypithecus geei*; fifth row: *Saguinus bicolor, Cebuella pygmaea, Cebus capucinus, Pithecia pithecia, Theropithecus gelada (infant), Alouatta seniculus*. (**b**) Some species, like *Macaca nigra* represented here, boast traits for which we lack adaptive explanations, like the temporal wedge. 1: Sclera; 2: conjunctiva; 3: black rings around the iris; 4: temporal wedge; 5: iris. All photographs are reproduced under CC license. *Hylobates pileatus* by Rhett Butler; *Theropithecus gelada, Gorilla gorilla gorilla, Theropithecus gelada (infant),* and *Macaca nigra* by Mogens Trolle.

Kobayashi and Kohshima’s seminal studies^1,2^ brought attention to the dramatic variation present in primate eye colour and morphology that was further explored by others^3–7^ (Fig. 1); nevertheless, two decades later our understanding of the drivers underlying said diversity remains poor. This diversity includes variation in shape of the eye outline, in conjunctival and iridial colouration, and in other traits for which we lack adaptive explanations or hypotheses^8^ (Fig. 2).

Because eyes are organs that mediate an organism’s interaction with its environment through light, investigation of external eye morphology in vertebrates has revolved around visual functions. Examples of these investigations include discovering a relationship between shape of the pupil and foraging mode in 214 species of land vertebrates^9^, and linking activity patterns^10^, foraging mode^11^, and habitat preference^12^ with eye shape in birds.

In contrast to the literature in other vertebrates, the focus of the study of external eye appearance in primates has been on suggested communicative functions, i.e., not how eyes see, but how they are seen by others. In early, influential work, Kobayashi and Kohshima proposed that t he contrast between the dark iris and light sclera evolved exclusively in humans to facilitate the perception of gazing by conspecifics, termed the “gaze signalling” hypothesis. These authors rejected alternative ecological explanations for variation in conjunctival depigmentation, such as darker conjunctiva functioning as an anti-glare mechanism^13^. Crucially, this anti-glare theory remains untested. The anti-glare theory proposes that eye pigmentation serves to prevent excessive scattered light and loss of contrast sensitivity due to intense light from the sun^14^. Face paints used by athletes have a similar anti-glare function^15^. In contrast, Kobayashi and Kohshima argued, all nonhuman primates evolved so-called ‘cryptic’ eye colouration in which the different sections of the eye are coloured in a way that hides gaze direction. This “gaze camouflage” hypothesis suggests that hiding gaze direction can function either to avoid intraspecific conflict triggered by the perception of eye contact, or to avoid predation^2^.

More recent studies with subsets of primate species do not support the rigid camouflage/signalling dichotomy in colouration in the eyes of humans and those of other primates, undermining the proposal that communicative functions drive variation in primate external eye appearance. Conjunctival depigmentation is sometimes apparent in gorillas^3^ . orangutans^4^, or bonobos^5^. Adding to this work, Caspar et al.^6^ concluded that the “gaze camouflage” and “gaze signalling” hypotheses^2^ could not explain patterns of pigmentation in hominoid primates due to the lack of correspondence between conspicuous eye colouration patterns and sophisticated reliance on eye gaze cues. Caspar et al.^16^ showed that hylobatids perform poorly in tasks for which they are required to follow eye-gaze (“glancing”), yet their eye colouration is as conspicuous as that of chimpanzees^6^. Similarly, Perea-García^4^ and Perea-García et al.^5^ showed that some great ape species display similar patterns of contrast between different eye parts to those of humans (e.g.: Sumatran orangutans, bonobos, and chimpanzees), yet Kaplan & Rogers^17^ showed that the gazing behaviour of Bornean and Sumatran orangutans and reliance on gaze cues appears reduced as compared to humans and can include pronounced gaze aversion .

The focus on communicative functions to explain variation in external eye appearance has resulted in a lack of exploration of the role of ecology in driving these patterns . Apart from the visual functions proposed by the anti-glare theory, which remains untested, darker conjunctivae could provide UV protection, as also provided by fur, hair, and skin, which become darker closer to the equator^18–20^). In addition, darker conjunctivae could provide protection against pathogens (Gloger’s rule^21^). Thus, ecological factors may have had a greater impact on the pigmentation of the conjunctiva than has been assumed.

The focus on external eye appearance and communicative functions has also led researchers to neglect variation in iris colour and its potential functions—even though primates feature some of the few mammalian species to display iris colours outside of brown^22–25^ (Fig. 2). Light coloured irises allow more light to enter the eyeball but also change the spectral quality of the light that hits the retina to a bluer hue, and blue light affects circadian rhythms in humans^26–28^. For instance blue irises have been associated with reduced incidence of seasonal affective disorder in people living far from the equator^29^. Latitudes farther from the equator receive less overall solar irradiance of all wavelengths with less short wavelength (blue) radiation^28^, and the circadian-rhythm hypothesis suggests that bluer irises in primates should be found at higher latitudes to maximise the incidence of blue light entering the eyeball^8^. Greater incidence of short-wave (blue) light results in the activation of intrinsically photosensitive retinal ganglion cells^27^ which, by suppressing melatonin release, increase wakefulness.

In sum, while there are diverse hypotheses about the factors driving variation in shape and colouration in human and other primates’ eyes, there is little support for many of them, and there is a need for a large comparative examination of how diverse ecological or social factors may impact the evolution of eye shape and colouration patterns in primates.

To assess variation in the shape and colouration of primate eyes, and to better understand where humans fit within this range, we measured the width-to-height ratio (WHR), sclera-size index (SSI), brightness of the iris, conjunctiva, and sclera, and hue of the iris in Hue Saturation Brightness (HSB^30^) in photographs of 77 anthropoid primate species. We used these quantitative measurements in combination with phylogenetically informed methods to test (a) whether horizontal elongation of the eye (WHR and SSI) increases with body mass and degree of terrestriality; (b) whether pigmentation in the conjunctiva has protective functions against glare or UV radiation, leading to more pigmented conjunctiva in species closer to the equator or to more pigmented conjunctiva in diurnal species; (c) whether irises reflect more blue light further away from the equator to compensate for reduced levels of blue light at these latitudes, and; (d) whether intrasexual competition leads to less irido-conjunctival contrast (to hamper the perception of eye contact by conspecifics, reducing antagonistic eye contact).

## Results

### Conjunctival depigmentation is a continuously varying trait across anthropoid primates

**Figure 3:**
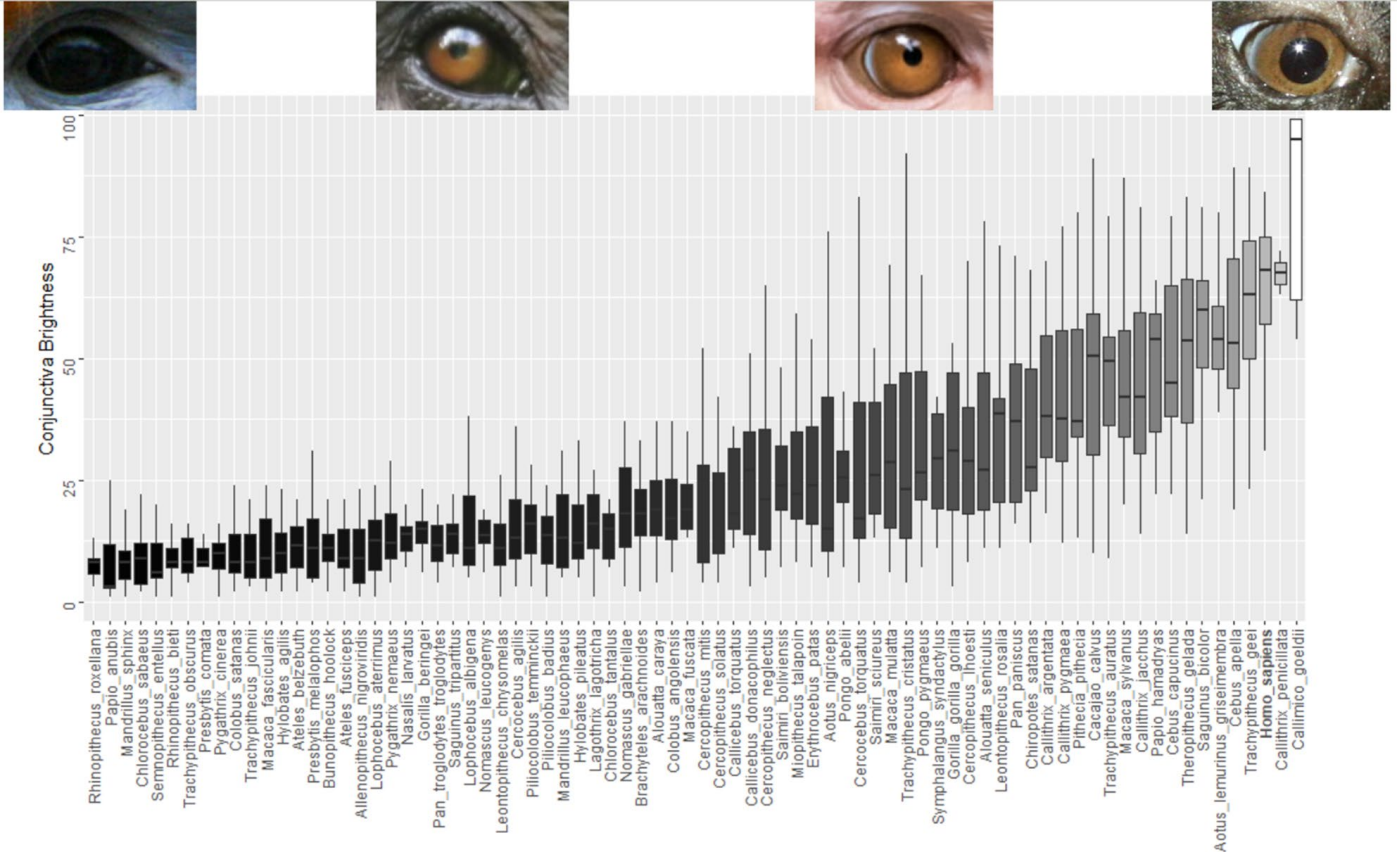
Conjunctival depigmentation is a continuously varying trait. Boxes are ordered according to the mean value. Lower and upper hinges represent the first and third quartiles. Middle bars represent median values. The two extremes (*R. roxellana* and *C. goeldii*) and two intermediate values (*P. troglodytes* and *M. mulatta*) are pictured above for reference.

To investigate variation in conjunctival pigmentation across species, and to test whether humans are distinctive in terms of the extent of depigmentation, we plotted the mean brightness of the conjunctiva across all species (Fig. 3). While humans are among the species with the least pigmented conjunctiva, at least two other species appear to display more depigmentation. Furthermore, there is no considerable gap between the values of humans and those of the adjacent species. To assess eye conspicuity as a function of contrast between iris and conjunctiva, we plotted the mean difference in measurements of brightness for each of our sampled species (Figure S1A). Humans had the third highest value, but again their mean (35.95 +/− 3.97) and range (10–75) were comparable to adjacent species (*Lophocebus albigena* and *Cercopithecus solatus*). Kobayashi and Kohshima^2^ observed that the portion of the sclera most distal to the iris was as depigmented as human sclerae in nonhuman primates. Our measurements show that this part of the eye also follows a gradient of pigmentation across species (Figure S1B), and that areas of sclerae distal to the iris are on average more depigmented than the conjunctiva (Wilcoxon test: W = 1812, p-value < 0.01).

### Eyes become more elongated with increases in body mass and terrestriality

Both measures of shape of the eye outline, SSI and WHR, were also continuously distributed across species. However, in both measurements, humans had the highest values. There was limited overlap in range between the lowest values of humans and the higher values of the closest species (*G. beringei* for SSI and *T. gelada* for WHR; Figure S2A).

We tested the hypothesis that scleral exposure increases with body size by using phylogenetic generalised least squares (PGLS) analysis, entering female body mass (in grams) as the independent variable and SSI as the dependent variable. PGLS incorporates phylogenetic information to correct for relatedness between species in our sample. Our data corroborated the suggested relationship between increased body mass and greater horizontal elongation of the eye outline, leading to more exposed sclera (SSI) (β = 0.11, SE = 0.09, t = 5.67, p < 0.01, n = 70).

To test whether horizontal elongation of the eye outline increases with degree of terrestriality, we classified our species as fully arboreal, semi-arboreal, or fully terrestrial and ran a phylogenetic ANOVA with WHR as the dependent variable. Like PGLS, phylogenetic ANOVA incorporates phylogenetic relatedness to correct for non-independence of our data points. Mean WHR values increased with degree of terrestriality (Fully arboreal: 1.72  ± 0.15; semi-arboreal: 1.90  ± 0.19; fully terrestrial: 2.28  ± 0.22; F = 10.78, p < 0.01). After correcting for multiple comparisons, the difference was significant between arboreal and terrestrial species: p < 0.01, and between semi-arboreal and terrestrial species: p = 0.04, but only neared significance between arboreal and semi-arboreal species: p = 0.07; Figure S2B).

### Global patterns of conjunctival pigmentation support photoprotective functions

**Figure 4:**
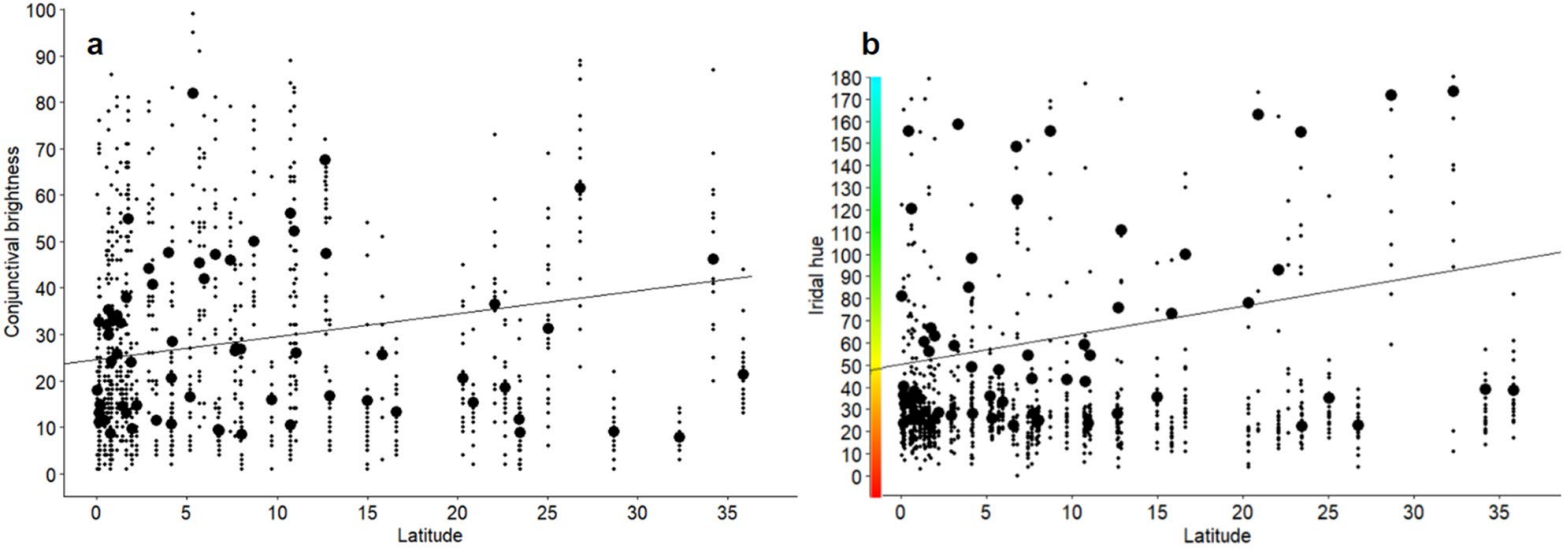
(**a**) Conjunctival brightness is greater in species whose native ranges are farther from the equator. (**b**) Iridal hue is greater in species whose native ranges are farther away from the equator. The Y axis represents hue in degrees (HSB) and chromatically. For both graphs, the regression line is pictured and small dots represent individual data points. Large dots represent species’ means.

To test the hypothesis that conjunctival pigmentation has photoprotective functions, we explored the relationship between conjunctival brightness and mean latitude of a species’ distribution (taken from^31^) with a PGLS. This analysis showed a significant positive association between distance from the equator and measurements of conjunctival brightness (β = 0.49, SE = 0.22, t = 2.29, p = 0.022, n = 61) (Fig. 4a). Following the same hypothesis, we also expected diurnal species to display greater pigmentation than nocturnal species. To test this prediction, we compared measurements of brightness of the conjunctiva of the nocturnal (*Aotus spp.*) and diurnal species in our sample with phylogenetic ANOVA but found no difference in their brightness (F = 0.85; p = 0.68, n = 70).

### Global patterns of iridal hue support circadian functions

To test whether the irises of anthropoid primates become bluer further from the equator, affecting the spectral quality of light reaching the retina , we ran a PGLS with latitude as the independent variable and iris hue as the dependent variable. On average, anthropoid irises displayed a hue of ~ 50º at the equator (orange-yellow, or ~ 600 nm), and hue increased with distance from the equator (β = 1.31, SE = 0.50, t = 2.60, p < 0.01, n = 62) up to a hue of ~ 110º at 35°N. (yellow-green, or ~ 550 nm) (Figs. 4b and S3).

### Patterns of conjunctival pigmentation do not support the gaze camouflage hypothesis

To test the gaze camouflage hypothesis we asked whether species with greater canine size sexual dimorphism (as a proxy of intraspecific male aggression^32^) had less irido-conjunctival contrast. Male intrasexual competition is a context in which we expect eye contact to be used as a means of establishing dominance^33,34^. We found no relationship between canine size dimorphism and irido-conjunctival contrast with a PGLS (β = 0.96, SE = 0.62, t = 1.55, p = 0.13, n = 52), suggesting that irido-conjunctival contrast is not related to ocular signalling in agonistic contexts.

## Discussion

Early studies suggested that humans were the only primate species with depigmented conjunctiva^1,2^, but here we showed that the difference in pigmentation of both the conjunctiva and sclera across anthropoid primate species—including us—is gradual. We also found continuity in patterns of irido-conjunctival contrast (e.g., average difference in measurements of brightness between iris and conjunctiva). These results are in line with recent examinations of orangutan^4^, gorilla^3^, chimpanzee and bonobo^5^, and Hominoid^6^ external eye appearance. We show here that the finding is generalizable to the broader taxon of anthropoid primates.

Using phylogenetically informed statistical methods, we showed that horizontal elongation of the eye outline is positively correlated to size and/or degree of terrestriality in primates. These results replicated Kobayashi and Kohshima’s original observations^1^. Our results also supported Mayhew and Gómez^3^ in concluding that the most distinct feature of human eyes compared to other primates is the degree to which they are horizontally elongated. All the species with the largest SSI, with the exception of *C. torquatus* and *T. gelada* (also land-living primates), are great apes with large body masses. One tree-living species, *Lophocebus aterrimus*, who also displays a large SSI, has recently adapted to tree-living but still retains skeletal adaptations for land-living^35^, and may retain land-adaptive eye shape as well. One small ape species, *S. syndactylus*, was the heaviest among the gibbons^36^ in our sample, and displayed the highest degree of horizontal elongation compared to other hylobatids in previous studies^6^, in line with expectations. Eye shape also differs between groups of species based on whether they were classified as fully arboreal, semiarboreal, or fully terrestrial, after correcting both for phylogenetic relatedness and multiple comparisons. Horizontal elongation of the eye outline may therefore constitute a widespread adaptation to terrestrial lifestyles that predisposes species to rely on conspecific ocular cues for inter-individual coordination. The present data did not allow us to disentangle the separate effects of size and terrestriality on eye shape because both body weight and terrestriality are related in primates, i.e., branches are less likely to support the body weight of heavier animals. Further investigations of body mass and horizontal eye elongation in other taxa will be necessary to tease apart the effect of body mass and terrestrial locomotion.

Our data supported the hypothesis that colouration features of the eyeball such as conjunctival pigmentation are related to differences in quantity and quality of light in the environment to which each species is native. To estimate the quality and quality of light in the environment we used latitude as a proxy, as it is well correlated with solar radiation globally^37^. Accordingly, we found that the conjunctivae of species living closer to the equator, with higher amounts of solar radiation in the environment, were more heavily pigmented. Other studies have shown latitude to be useful in predicting pigmentation levels in skin^38^, fur^18^, and feathers^21^. However, alternative explanations for increased pigmentation closer to the equator refer to antibacterial functions in more humid environments^21^. Even though the difference between nocturnal and diurnal species in our sample was not significant, the nocturnal subsample consisted only of two species, so we recommend expanding this group in future investigations. Future finer grained studies should also examine other predictors of overall light levels such as habitat openness, which has been shown to affect eye structures in owls^12^. For example, callitrichids and atelids living in the same range will be differentially exposed to UV because atelids tend to be high-canopy dwellers while callitrichids stay in the lower canopy, often living inside hollow trees.

Our results suggested that species with bluer irises live in environments with relatively less amounts of blue light. Irises at the equator are orange-yellow, turning bluer with increasing distance from the equator. The finding that irises reflect more short-wavelengths farther from the equator constitutes a case of convergent evolution across distantly related anthropoid taxa. This finding suggests an important role of natural selection in the emergence of blue irises in northern human populations as well as in other primates. For example, Japanese macaques (*Macaca fuscata*) are the most northern-living nonhuman primates and have striking green-to-blue irises that become bluer in islands more towards the north^22^. Beyond image-forming functions, the eye retina contains photo-sensitive cells that regulate circadian functions based on the availability of short wavelength (blue) light^27,39,40^. Because blue light is scarcer in northern latitudes^28^, changing the structural properties of the iris so that it scatters more blue light into the retina could compensate for this scarcity. In humans, observational evidence shows that among individuals suffering from seasonal affective disorder (SAD) living far from the equator, those with blue eyes are less depressed and fatigued than those with dark eyes^29,41^. In addition, experimental evidence demonstrates that increased levels of blue light promote wakefulness in humans^42^.

Because the results we present support a general effect of spectral quality in patterns of ocular pigmentation and colouration, it should follow that similar effects are found in species with large ranges extending North–South, or experiencing important seasonal changes in lighting conditions. However, the literature on adaptive functions of eye colouration is surprisingly scarce^43^, especially outside of primates^44^. The scarcity of investigations into the adaptiveness of variation in external eye appearance makes it hard to compare our results with patterns in other taxa. Nonetheless, there is evidence supporting the more general idea that the external appearance of eyes differs depending on the typical spectral qualities of a species. For example, the tapetum lucidum of reindeer changes seasonally to adapt to the stark differences in lighting conditions typical of high latitudes^45^. In passerine birds, iris colour has been suggested to respond to camouflage functions^43^ while communicative functions have been proposed in the Nile tilapia^44^.

Zooming in on species-specific functions of primate external eye appearance may reveal exceptions in the patterns we found at the infraorder level. For instance, selective factors, other than blue light availability, have been proposed to explain why specific species of lemur and spider monkey in more equatorial distributions have blue eyes^23,24^. In these species, blue eye colour is proposed to have evolved in response to sexual selection, or as a reinforcement signal to prevent hybridization between closely related species^24^, similar to the proposed functions of patterns of facial appearance in guenons^46^. While the predominant explanation for blue eyes in humans revolves around sexual selection^47^, our comparative data suggests that natural selection may act on iris colour across primates to minimize loss of exposure to short wavelengths of light at higher latitudes^8^.

To test the cryptic gaze hypothesis, we used the average difference in measurements of brightness of the iris and surrounding conjunctiva as a proxy for eye saliency. We could not test the relationship between predation and irido-conjunctival contrast because consistent predation data for primate species are lacking^48^. However, to test the hypothesis in conspecific signalling, we investigated whether irido-conjunctival contrast was lower in species with greater canine size dimorphism. Canine size dimorphism is a reliable correlate of male intrasexual competition—a context in which staring can be used to express dominance through eye contact^32,33^, and where subordinate individuals would benefit from hampering the visibility of their gaze direction (as proposed by Kobayashi & Kohshima^1^). We found no relationship between canine size dimorphism and irido-conjunctival contrast. Our results do not support an adaptive advantage for primates, as a taxon, to hamper the transmission of social gaze information between conspecifics in agonistic interactions, in line with the observation that nonhuman primates appear to readily detect gaze^49^. Furthermore, recent models^50^ as well as experimental evidence^51^ support the proposal that hyperpigmented conjunctiva (like those of chimpanzees) may contribute to a salient eye direction if the iris is bright^5^, owing to the contrast between adjacent tissues. Anthropoid primates are highly visual animals^52^ that rely on attention structure (e.g., who looks at whom^53^) to infer hierarchy. Doing so could enable subordinate individuals to avoid costly conflict by inferring the goals and intentions of more dominant individuals^54^. It seems thus improbable that the entire taxon—with the exception of humans—evolved to have a cryptic gaze.

In conclusion, we show that external eye shape in primates is related to physical constraints and eye colour is more related to ecological factors than to communicative functions. Caspar et al.^6^ also found that patterns of irido-conjunctival contrast at the superfamily (Hominoid) level do not correlate with socio-cognitive functions. Adaptations affecting external eye appearance to enhance communicative functions are unlikely to disrupt adaptations that enhance vision or photoprotection of the eyes, especially in highly visual animals like primates.

## Methods

### Taxonomic samples

The photographs we measured come from multiple sources (publicly available photographs on the internet, n = 1536), as well as photographs we purchased from professional wildlife photographer Mogens Trolle (n = 76), and photographs that were taken by Mariska Kret (n = 19) at the Apenheul Primate Park. We included photographs in our sample only if the different parts of the eye were distinguishable. We avoided photographs that were obviously edited, over, or underexposed. Our samples included anthropoid species (Old World monkeys and apes and New World monkeys) (N = 77). We limited our focus to anthropoid species because prosimians are all arboreal and have eyes with little exposed conjunctiva^1^. We aimed to include two species per genus, including the most basal and derived species. Sometimes this was not possible due to lack of materials. In that case we either found the next most derived species in the taxon, or we dropped the branch (representing that genus) if no more species were available. Due to the possibility that our measurements could reflect differences in lighting conditions apart from real differences across species, we followed the recommendations by Laitly et al.^55^ regarding the use of uncalibrated images in colour research to include 12–14 samples per species. We aimed to collect at least 20 photographs of different adult individuals per species but, in some cases, this was not possible (n = 6). We identified individuals whenever possible to avoid sampling the same one twice. When it was not clear whether the animal had already been sampled, we omitted the photograph. Links to the publicly available photographs are available upon request.

### Eye measurements

For each photograph, we sampled the eye on the left of the photograph. If that eye showed confounding factors (stark shadows, specular reflections), or was not pictured in full frontal angle for eye shape measurements, we used the right eye. We measured our images with the PAT-GEOM plugin for ImageJ^56^. We measured width-to-height (WHR), sclera size index (SSI)^1^, iridial, conjunctival, and scleral brightness. WHR and SSI were measured only in photographs without obvious facial expressions, and in which the photographs were frontal (i.e., both ears, eyes, and nostrils were visible). The length of the sampling square for the conjunctiva was determined by the SSI of each individual. If the SSI < 1.5 then the conjunctiva ended at 1/3 the length of the iris radius (or until reaching confounding factors such as eye outline) into the conjunctiva. If SSI =  > 1.5 then the conjunctiva ended at 2/3 the length of the iris radius (or until reaching confounding factors such as eye outline or specular reflections) towards the posterior eyeball. All distances were measured in pixels. This is not an issue for ratio-based measurements such as WHR and SSI. The iris was measured from the edge of the pupil towards the edge of the iris, or before reaching confounding factors such as specular reflections, temporal wedges, or other features of the eye described in Perea-García et al.^8^. We also noted the presence or absence of three traits that may aid observers in discerning gaze direction or other socially relevant information as described in Perea-García et al.^8^: black rings or white rings around the iris, and a temporal wedge (Fig. 2).

### Statistics and reproducibility

Because phenotypic traits are influenced not only by adaptation, but also by past evolutionary history, we used phylogenetic generalised least squares (PGLS) and phylogenetic ANOVA—methods that incorporate information about phylogenetic relatedness to account for non-independence of data points between the species in our sample^57^. Ocular traits (WHR, SSI, Conjunctival Brightness, Iris Hue, and average difference in measurements of brightness between iris and conjunctiva) were used as response variables in separate regression models (PGLS) or phylogenetic ANOVAs. Canine Size Dimorphism, Female Body Mass and Latitude were used as independent variables. The choice to use Female Body Mass across species was taken to minimize potential sex-effects from including sexually dimorphic species. For the latitude data, we first made the values absolute to express distance from the equator. For PGLS, we examined diagnostic plots with the plot.pgls function in the caper package, and applied Tukey transformation to variables if the transformation improved the homoscedasticity of residuals. For phylogenetic ANOVA, we tested the residuals of response variables for normality with Shapiro–Wilk tests, and applied Tuk transformation when variables were not normally distributed. The two nocturnal species in our sample (*Aotus spp.*) were removed from analyses relating eye traits with photopic drivers (iris hue and conjunctival pigmentation over distance from the equator). Phylogenetic analyses vary in the number of species, depending on available data for each variable and species, as well as whether they were present in our phylogenetic tree block (*n* for each analysis is indicated in the *Results* section). The inclusion of *Homo sapiens* in our measurements enabled qualitative comparisons but we did not include *Homo sapiens* in phylogenetic analyses (both PGLS and phylogenetic ANOVA) because of the species’ global distribution and extremely high intraspecific phenotypic variation. All analyses were conducted in R (4.0.3). For phylogenetic ANOVA we used the phylANOVA function from the phytools R package^58^. We used the pgls function with Pagel’s *lambda* correlation structure^59^ from the caper package in R^60^ for our PGLS analyses. The consensus tree in Figure S3 was drawn using Mesquite. Graphs were drawn using the ggplot2 package^61^.

### Phylogenetic data, anatomic, social, and ecological variables

We used a consensus tree built from a combination of molecular data from the 10kTrees project (https://10ktrees.nunn-lab.org/) to account for phylogeny in our PGLS and phylogenetic ANOVAs. Our variables were taken from the supplementary materials of Kamilar and Cooper^35^. These variables are Sexual Dimorphism in Canine Size, a reliable proxy of male intrasexual competition in anthropoid primates^36^; Female Body Mass, an average of the weight (in kilograms) of females in a given species (we preferred female averages to avoid inconsistencies stemming from stark differences in body weight between sexes in dimorphic species); Latitude (expressed as the absolute degrees away from the equator for the centroid of each species’ distribution), and; Activity Patterns (whether the species is predominantly active during day or night time). We scored species’ degree of terrestriality with information from Williamson et al.^62^ and the Animal Diversity Web.

## Supplementary Information

Supplementary Information.

## Data Availability

The datasets generated and/or analysed during the current study are available in the OSF repository, https://osf.io/ms4ve/?view_only=fe22c39827ad42b6b1747d101f811fbc. Raw photographic samples are available upon request.
