# Supplementary material for: Ecological factors are likely drivers of eye shape and colour pattern variations across anthropoid primates
Source: Sci Rep. 2022 Oct 15;12:17240. doi: 10.1038/s41598-022-20900-6 (PMC9569326; doi:10.1038/s41598-022-20900-6)
Supplement: Supplementary file 1 — Supplementary Information. [file 41598_2022_20900_MOESM1_ESM.docx]

**Supplementary materials**

**Table S1.-** Mean values for all our measurements. WHR: width-to-height ratio of the eye outline. SSI: Sclera size index. PupilB: pupil brightness in HSB colour space. IRH: Iris hue in HSB. IRB: Iris brightness in HSB. TempWedge: Whether temporal wedge was visible in the majority of our samples. BlackRings: Whether a dark ring around the iris was visible in the majority of our samples. WhiteRings: Whether an white ring was visible in the majority of our samples. ConjunctB: Conjunctiva Brightness in HSB. SB: Sclera Brightness in HSB. Contrast: absolute difference between ConjunctB and IRB in Type 1, and between IRB and ConjunctB in Type 2. pupContrast: absolute difference between IRB and PupilB. Type: Whether the conjunctiva is lighter than the iris (Type 1), or vice-versa (Type 2).

| **Species** | **WHR** | **SSI** | **PupilB** | **IRH** | **IRB** | **WhiteRings** | **TempWedge** | **BlackRings** | **ConjunctB** | **SB** | **Type** | **Contrast** | **pupContrast** |
| --- | --- | --- | --- | --- | --- | --- | --- | --- | --- | --- | --- | --- | --- |
| Allenopithecus_nigroviridis | 1.62 | 1.43 | 10.2 | 23.6 | 60.6 | No | Yes | No | 13.0 | 10.5 | 2.0 | 47.6 | 50.3 |
| Alouatta_caraya | 1.66 | 1.30 | 13.5 | 78.0 | 28.4 | No | No | Yes | 20.5 | 30.4 | 2.0 | 10.6 | 14.9 |
| Alouatta_seniculus | 1.81 | 1.41 | 14.9 | 34.5 | 35.3 | No | Yes | Yes | 35.2 | 45.6 | 1.0 | 10.2 | 20.4 |
| Aotus_lemurinus_griseimembra | 1.43 | 0.99 | 15.9 | 47.1 | 49.2 | No | No | Yes | 55.4 | 0.0 | 1.0 | 12.3 | 34.5 |
| Aotus_nigriceps | 1.46 | 1.14 | 11.8 | 28.0 | 45.3 | No | No | No | 26.9 | NA | 2.0 | 22.6 | 33.6 |
| Ateles_belzebuth | 1.80 | 1.31 | 14.5 | 155.5 | 23.2 | Yes | No | No | 11.4 | 14.5 | 2.0 | 13.1 | 8.8 |
| Ateles_fusciceps | 1.81 | 1.50 | 13.7 | 128.5 | 19.0 | Yes | No | No | 11.8 | 8.7 | 2.0 | 8.7 | 6.1 |
| Brachyteles_arachnoides | 1.88 | 1.42 | 13.0 | 139.0 | 18.9 | No | No | No | 15.3 | 20.7 | 2.0 | 5.8 | 6.0 |
| Bunopithecus_hoolock | 1.63 | 1.29 | 12.4 | 155.1 | 21.2 | No | No | No | 11.7 | NA | 2.0 | 9.8 | 8.8 |
| Cacajao_calvus | 1.76 | 1.20 | 9.1 | 47.9 | 32.3 | No | Yes | Yes | 45.3 | 48.0 | 1.0 | 15.5 | 22.6 |
| Callicebus_donacophilus | 1.70 | 1.15 | 13.6 | 73.1 | 33.9 | No | No | Yes | 25.6 | 34.0 | 1.0 | 12.3 | 20.3 |
| Callicebus_torquatus | 1.72 | 1.20 | 15.8 | 71.4 | 40.5 | No | Yes | No | 25.4 | 16.0 | 2.0 | 20.8 | 24.3 |
| Callimico_goeldii | 1.68 | 1.07 | 14.1 | 25.9 | 62.3 | No | Yes | Yes | 81.8 | NA | 1.0 | 23.0 | 48.1 |
| Callithrix_argentata | 1.72 | 1.07 | 8.2 | 58.5 | 32.0 | No | No | No | 40.8 | NA | 1.0 | 12.3 | 23.8 |
| Callithrix_jacchus | 1.78 | 1.06 | 5.4 | 22.7 | 48.8 | No | No | No | 47.2 | NA | 2.0 | 10.4 | 43.7 |
| Callithrix_penicillata | 1.62 | 0.96 | 10.5 | 27.8 | 54.1 | No | Yes | Yes | 67.5 | NA | 2.0 | 6.0 | 43.6 |
| Callithrix_pygmaea | 1.54 | 1.10 | 5.7 | 33.4 | 52.4 | No | No | Yes | 41.8 | NA | 2.0 | 15.7 | 46.7 |
| Cebus apella | 1.81 | 1.39 | 14.3 | 42.3 | 41.9 | No | No | Yes | 56.1 | 67.4 | 1.0 | 19.2 | 27.7 |
| Cebus capucinus | 1.49 | 1.42 | 10.0 | 104.0 | 21.1 | No | No | Yes | 42.6 | 45.0 | 1.0 | 24.3 | 11.2 |
| Cebus_capucinus | 1.54 | 1.16 | 10.1 | 155.7 | 20.6 | No | Yes | Yes | 50.1 | 64.6 | 1.0 | 34.0 | 10.5 |
| Cercocebus_agilis | 1.90 | 1.38 | 11.1 | 83.4 | 34.3 | No | Yes | No | 15.7 | 32.5 | 2.0 | 21.0 | 23.2 |
| Cercocebus_torquatus | 1.85 | 1.72 | 8.7 | 27.8 | 26.9 | No | Yes | Yes | 28.5 | 47.4 | 2.0 | 20.0 | 19.3 |
| Cercopithecus_lhoesti | 1.68 | 1.50 | 16.8 | 34.7 | 54.8 | No | Yes | Yes | 34.0 | 64.0 | 2.0 | 23.1 | 38.0 |
| Cercopithecus_mitis | 1.81 | 1.20 | 13.5 | 25.3 | 48.5 | No | No | Yes | 24.0 | 47.3 | 2.0 | 26.2 | 35.0 |
| Cercopithecus_neglectus | 1.69 | 1.30 | 8.2 | 24.9 | 30.4 | No | Yes | Yes | 25.8 | 36.7 | 2.0 | 12.2 | 22.6 |
| Cercopithecus_solatus | 1.77 | 1.47 | 22.6 | 38.0 | 55.9 | No | Yes | No | 24.1 | 95.0 | 2.0 | 41.5 | 33.8 |
| Chiropotes_satanas | 1.65 | 1.44 | 14.3 | 60.9 | 37.9 | No | Yes | No | 38.1 | 20.0 | 2.0 | 10.6 | 24.8 |
| Chlorocebus_sabaeus | 1.65 | 1.19 | 7.6 | 25.8 | 35.9 | No | Yes | No | 8.7 | 73.0 | 2.0 | 26.9 | 28.3 |
| Chlorocebus_tantalus | 1.82 | 1.28 | 12.0 | 85.3 | 25.6 | No | Yes | No | 18.3 | 15.0 | 2.0 | 14.1 | 13.6 |
| Colobus_angolensis | 1.77 | 1.21 | 9.6 | 98.1 | 16.8 | No | No | No | 20.6 | 19.7 | 1.0 | 10.8 | 7.3 |
| Colobus_satanas | 1.87 | 1.28 | 10.7 | 63.0 | 23.3 | Yes | No | No | 9.6 | 13.5 | 2.0 | 13.8 | 12.7 |
| Erythrocebus_patas | 1.73 | 1.31 | 8.4 | 24.2 | 30.0 | No | Yes | Yes | 26.8 | 36.0 | 2.0 | 7.6 | 22.3 |
| Gorilla_beringei | 2.02 | 1.89 | 13.3 | 35.9 | 38.6 | No | No | No | 14.7 | 27.0 | 2.0 | 25.0 | 25.4 |
| Gorilla_gorilla_gorilla | 2.06 | 1.72 | 10.3 | 27.4 | 36.8 | No | No | Yes | 32.9 | 40.1 | 1.0 | 23.9 | 26.3 |
| Homo_sapiens | 3.46 | 2.09 | 11.7 | 51.0 | 27.5 | No | No | Yes | 63.5 | 61.6 | 1.0 | 36.0 | 16.2 |
| Hylobates_agilis | 1.65 | 1.32 | 11.6 | 36.4 | 34.0 | No | No | No | 11.0 | 12.0 | 2.0 | 22.2 | 22.2 |
| Hylobates_pileatus | 1.60 | 1.22 | 18.6 | 110.9 | 22.9 | No | No | No | 16.7 | NA | 2.0 | 5.6 | 6.8 |
| Lagothrix_lagotricha | 2.00 | 1.34 | 13.0 | 80.9 | 22.8 | No | No | No | 17.9 | 32.3 | 2.0 | 7.8 | 9.8 |
| Leontopithecus_chrysomelas | 1.73 | 1.23 | 8.5 | 35.5 | 30.1 | No | Yes | No | 15.7 | NA | 2.0 | 20.5 | 21.5 |
| Leontopithecus_rosalia | 1.72 | 1.26 | 11.9 | 92.9 | 30.9 | No | No | No | 36.4 | 69.5 | 2.0 | 14.3 | 19.0 |
| Lophocebus_albigena | 1.84 | 1.48 | 15.3 | 32.6 | 45.8 | No | No | No | 14.9 | 16.7 | 2.0 | 35.4 | 29.7 |
| Lophocebus_aterrimus | 2.18 | 1.38 | 12.4 | 23.3 | 43.7 | No | No | No | 13.0 | 17.0 | 2.0 | 30.7 | 31.3 |
| Macaca_fascicularis | 2.03 | 1.52 | 7.2 | 49.0 | 44.6 | No | No | No | 10.5 | 14.2 | 2.0 | 34.3 | 37.4 |
| Macaca_fuscata | 1.84 | 1.37 | 12.4 | 38.3 | 38.3 | Yes | No | Yes | 21.3 | 35.9 | 2.0 | 17.0 | 25.9 |
| Macaca_mulatta | 1.81 | 1.38 | 7.5 | 35.1 | 37.9 | No | Yes | Yes | 31.1 | 30.2 | 2.0 | 14.3 | 30.3 |
| Macaca_sylvanus | 1.96 | 1.57 | 6.8 | 39.0 | 31.1 | No | No | Yes | 46.2 | 50.4 | 1.0 | 17.9 | 24.3 |
| Mandrillus_leucophaeus | 1.99 | 1.43 | 11.4 | 35.9 | 37.8 | No | Yes | No | 16.5 | 29.6 | 2.0 | 21.4 | 26.5 |
| Mandrillus_sphinx | 2.15 | 1.58 | 8.9 | 38.1 | 41.8 | No | Yes | No | 8.7 | 13.9 | 2.0 | 33.1 | 32.9 |
| Miopithecus_talapoin | 1.69 | 1.16 | 12.3 | 43.8 | 37.2 | No | No | Yes | 26.3 | 34.8 | 2.0 | 15.5 | 25.8 |
| Nasalis_larvatus | 1.74 | 1.18 | 7.3 | 28.8 | 25.9 | Yes | No | No | 14.4 | 10.0 | 2.0 | 10.8 | 18.6 |
| Nomascus_gabriellae | 1.53 | 1.22 | 18.4 | 178.9 | 20.3 | No | No | No | 18.4 | 39.0 | 2.0 | 5.6 | 2.8 |
| Nomascus_leucogenys | 1.72 | 1.28 | 13.7 | 162.8 | 18.7 | Yes | No | No | 15.2 | 17.7 | 2.0 | 4.6 | 5.6 |
| Pan_paniscus | 1.87 | 1.61 | 11.2 | 55.9 | 24.3 | Yes | No | Yes | 38.0 | 52.6 | 1.0 | 16.9 | 13.2 |
| Pan_troglodytes_troglodytes | 1.93 | 1.62 | 19.0 | 28.3 | 41.6 | No | No | No | 14.7 | 23.5 | 2.0 | 28.9 | 22.6 |
| Papio_anubis | 2.20 | 1.52 | 7.8 | 24.7 | 40.9 | No | No | No | 8.4 | 18.0 | 2.0 | 32.5 | 33.1 |
| Papio_hamadryas | 2.13 | 1.52 | 8.5 | 75.5 | 35.2 | No | No | No | 47.4 | 52.7 | 1.0 | 15.4 | 26.7 |
| Piliocolobus badius | 1.59 | 1.08 | 17.3 | 43.3 | 35.6 | No | No | No | 15.9 | 19.0 | 2.0 | 22.1 | 18.3 |
| Piliocolobus temminckii | 1.61 | 1.14 | 14.1 | 26.6 | 41.4 | No | Yes | No | 15.7 | 27.8 | 2.0 | 26.3 | 27.3 |
| Pithecia_pithecia | 1.69 | 1.28 | 14.1 | 27.0 | 38.0 | No | Yes | Yes | 44.1 | 72.7 | 1.0 | 12.9 | 23.9 |
| Pongo_abelii | 1.96 | 1.68 | 12.9 | 54.5 | 27.2 | No | No | Yes | 27.0 | 44.9 | 1.0 | 12.4 | 14.8 |
| Pongo_pygmaeus | 1.97 | 1.67 | 12.3 | 60.6 | 25.8 | Yes | No | No | 32.4 | 38.4 | 1.0 | 8.1 | 13.8 |
| Presbytis_comata | 1.62 | 1.10 | 12.7 | 148.6 | 18.4 | No | Yes | No | 9.3 | NA | 2.0 | 9.7 | 6.7 |
| Presbytis_melalophos | 1.62 | 1.24 | 13.2 | 158.7 | 16.6 | No | No | No | 11.5 | 35.0 | 2.0 | 5.0 | 4.2 |
| Pygathrix_cinerea | 1.72 | 1.24 | 10.0 | 129.5 | 13.7 | No | No | No | 9.4 | 23.0 | 2.0 | 4.4 | 3.9 |
| Pygathrix_nemaeus | 1.71 | 1.18 | 13.5 | 99.8 | 20.9 | Yes | No | No | 13.3 | 28.0 | 2.0 | 6.8 | 7.5 |
| Rhinopithecus_bieti | 1.86 | 1.26 | 7.0 | 171.7 | 7.2 | Yes | No | No | 9.1 | 17.0 | 1.0 | 2.7 | 0.9 |
| Rhinopithecus_roxellana | 1.94 | 1.30 | 5.4 | 173.7 | 8.0 | Yes | No | No | 7.9 | 7.5 | 1.0 | 2.2 | 2.7 |
| Saguinus_bicolor | 1.70 | 1.22 | 4.6 | 66.7 | 25.2 | No | No | No | 54.9 | NA | 1.0 | 32.9 | 20.7 |
| Saguinus_tripartitus | 1.59 | 1.14 | 14.2 | 104.8 | 32.4 | No | Yes | No | 14.8 | NA | 2.0 | 20.6 | 18.5 |
| Saimiri_boliviensis | 1.64 | 1.24 | 9.2 | 54.1 | 27.0 | No | No | Yes | 25.9 | 23.0 | 1.0 | 9.5 | 17.9 |
| Saimiri_sciureus | 1.68 | 1.11 | 8.9 | 120.3 | 12.8 | No | No | No | 29.8 | 56.0 | 1.0 | 14.9 | 3.9 |
| Semnopithecus_entellus | 1.67 | 1.24 | 9.9 | 22.1 | 30.9 | No | Yes | No | 8.8 | NA | 2.0 | 22.5 | 21.0 |
| Symphalangus_syndactylus | 1.69 | 1.61 | 15.7 | 40.2 | 34.3 | Yes | No | Yes | 32.7 | 41.6 | 2.0 | 12.1 | 22.2 |
| Theropithecus_gelada | 2.44 | 1.64 | 14.8 | 23.8 | 58.2 | No | Yes | Yes | 52.1 | 57.2 | 2.0 | 9.7 | 43.4 |
| Trachypithecus_auratus | 1.49 | 1.19 | 8.7 | 54.4 | 26.5 | No | Yes | Yes | 45.9 | 79.0 | 1.0 | 26.0 | 18.4 |
| Trachypithecus_cristatus | 1.58 | 1.19 | 14.4 | 25.6 | 36.9 | No | No | No | 31.9 | 52.3 | 2.0 | 20.2 | 22.6 |
| Trachypithecus_geei | 1.63 | 1.36 | 7.7 | 22.7 | 41.5 | No | Yes | Yes | 61.4 | 52.7 | 1.0 | 25.9 | 33.9 |
| Trachypithecus_johnii | 1.62 | 1.28 | 14.0 | 59.3 | 38.6 | No | Yes | No | 10.4 | NA | 2.0 | 27.8 | 25.6 |
| Trachypithecus_obscurus | 1.70 | 1.20 | 10.8 | 124.4 | 16.1 | No | Yes | No | 9.2 | 56.0 | 2.0 | 7.1 | 5.8 |


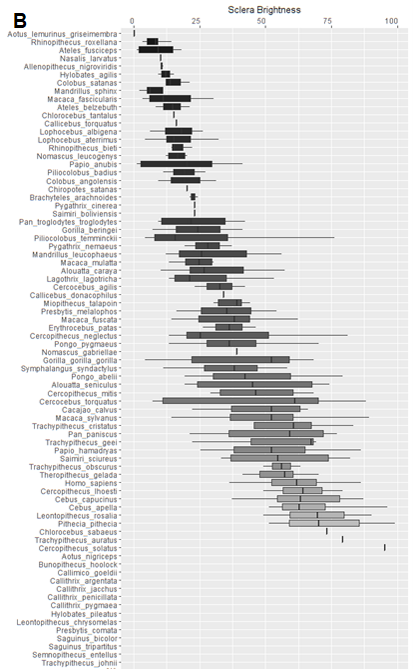

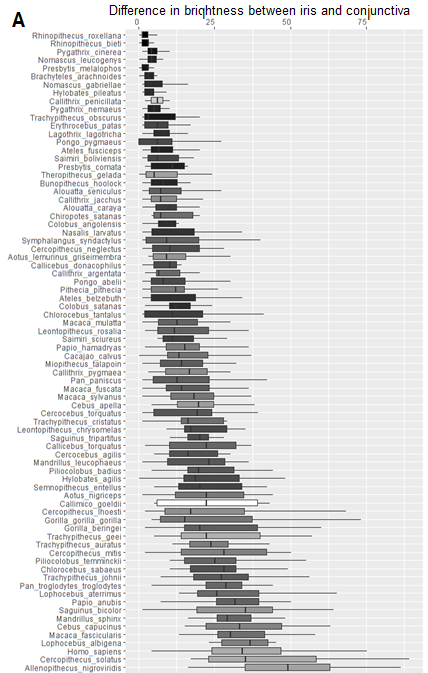


**Figure S1**.- A) Brightness of the portion of the sclera not covered by the conjunctiva (more distal from the iris) by species. Species with one vertical line had only one data point for this trait. We did not have photos of the sclera of species with empty rows. B) Absolute difference between the brightness of the iris and adjacent conjunctiva. Boxes are ordered according to the mean value. Values along the error bars represent the first and third quartiles. Middle bars represent median values.


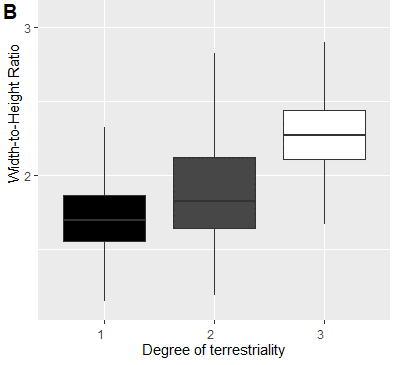

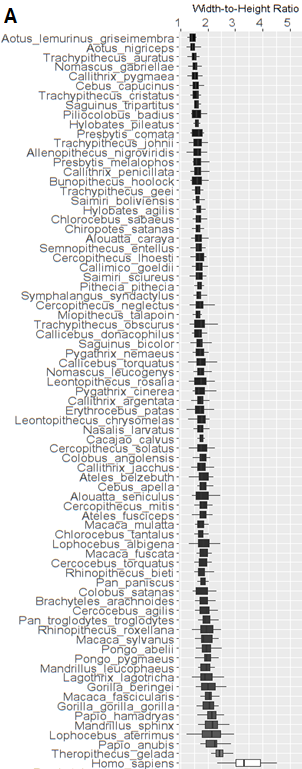


**Figure S2**.- A) WHR by species. B) WHR by degree of terrestriality. Boxes are ordered according to mean WHR value. Lower and upper values in the error bars represent the first and third quartiles for each category. Middle bars represent median values for each category.


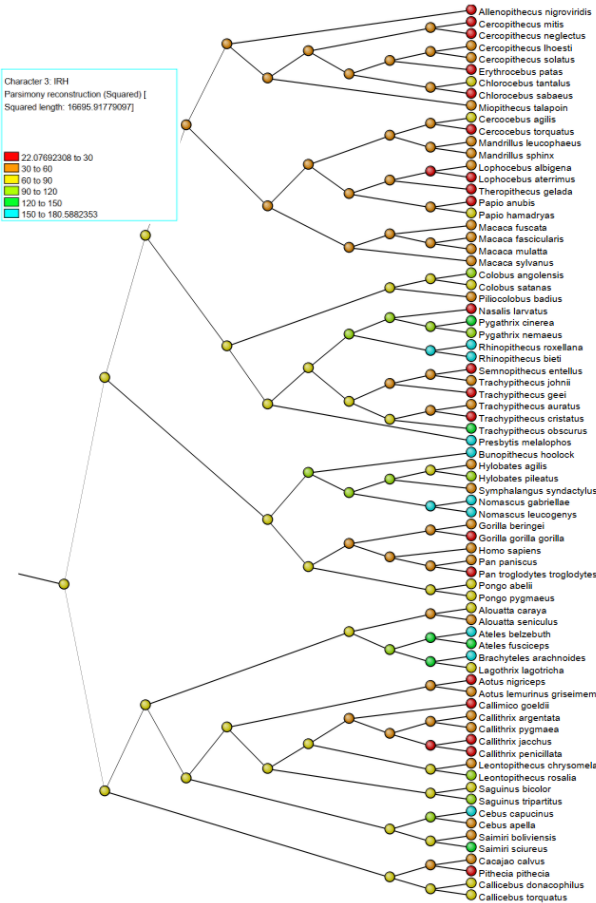


**Figure S3**.- Mean iris hue values (measured in HSB: Smith, 1978) per species, and parsimony reconstruction of ancestral states. The colours are binned in an approximation of actual hues.
